# Comparing the Efficacy of Ziziphus jujuba L. Product and Polyethylene Glycol in Elderly People with Functional Constipation: A Single-Blind Randomized Clinical Trial: Short title: Ziziphus jujuba L. and PEG in Elderly Constipation

**DOI:** 10.31661/gmj.v15i.4253

**Published:** 2026-09-18

**Authors:** Fereshte Fathi, Maryam Taghavi Shirazi, Ali ghobadi, Hosseinnataj Abolfazl, Assie Jokar, Shahram Agah

**Affiliations:** ^1^ Student Research Committee, Mazandaran University of Medical Sciences, Sari, Iran; ^2^ Institute for Studies in Medical History, Persian and Complementary Medicine, Iran University of Medical Sciences, Tehran, Iran; ^3^ Department of Traditional Medicine, School of Traditional Medicine, Iran University of Medical Sciences, Tehran, Iran; ^4^ School of Persian Medicine, Iran University of Medical Sciences, Tehran, Iran; ^5^ Department of Biostatistics and Epidemiology, Faculty of health, Mazandaran University of Medical Sciences, Sari, Iran; ^6^ Persian Medicine Faculty, Traditional and Complementary Medicine Research Center, Addiction Institute, Mazandaran University of Medical Sciences, Sari, Iran; ^7^ Colorectal Research Center, Iran University of Medical Sciences, Tehran, Iran

**Keywords:** Constipation, Ziziphus jujuba, Polyethylene Glycol, Clinical Trial, Elderly

## Abstract

**Background:**

Constipation is one of the most common gastrointestinal disorders, especially among the elderly. The tendency to use food-based remedies is increasing due to their safety and availability. Jujube is a plant recommended for treating constipation. this study was designed to compare the effectiveness of Jujube paste with polyethylene glycol (PEG) in elderly patients with functional constipation (FC).

**Materials and Methods:**

This single-blind, randomized, active-controlled clinical trial was conducted with 60 elderly individuals aged 60 years or older who had FC. Participants were randomly assigned to either the Jujube paste group (two tablespoons twice daily) or the PEG group (10-20 g/day) and underwent treatment for 4 weeks. Constipation symptoms and stool consistency, as primary outcomes, were assessed using the Rome IV criteria and the Bristol Stool Scale, respectively. Additionally, quality of life, acceptance, and satisfaction were measured.

**Results:**

At the end of the study, 26 participants in the PEG group and 25 in the Jujube paste group completed the trial. When comparing each group to their respective baseline, all clinical constipation symptoms, as defined by Rome IV criteria, showed significant improvement in both groups (P ≤ 0.002). Defecation frequency improved in both groups, with a significantly greater increase in the PEG group. This between-group difference remained significant after adjustment for baseline defecation frequency and education level (adjusted mean difference, 5.20 bowel movements/week; 95% CI, 1.53–8.87; P = 0.006). Quality of life improved in both groups, with a greater improvement observed in the Jujube group. Acceptance and satisfaction rates were significantly higher in the Jujube group compared to the PEG group (P < 0.05).

**Conclusion:**

Jujube paste may improve constipation symptoms in elderly patients with functional constipation and showed higher acceptance and satisfaction than PEG in this short-term trial. Larger, longer, and preferably blinded trials are needed to confirm efficacy.

## Introduction

Functional constipation (FC) is one of the most common bowel disorders in the general population, especially among the elderly.[1] Its prevalence increases with age, and there is a gender difference, with women experiencing it more often [1]. The self-reported prevalence of constipation in the elderly is estimated to be about 26% in women and 16% in men [1]. The number of people with constipation is projected to grow in the future [2]. In clinical practice, it is often not seen as a serious problem, but if left untreated, constipation can lead to fecal incontinence, fecal impaction, hemorrhoids, and fissures [3, 4], as well as worsen the prognosis of other conditions such as cardiovascular disorders and overall survival, significantly impairing quality of life (QOL) [5, 6].

Constipation can cause pain and bloating, but it is usually diagnosed based on symptoms like fewer than three spontaneous bowel movements per week, difficulty during defecation, lumpy or hard stools, a feeling of anorectal blockage, and incomplete evacuation [7]. A variety of risk factors, including genetic and lifestyle aspects, can contribute to constipation [8]. However, in the elderly, specific factors such as reduced social activity, psychological issues, aging of the pelvic floor, co-morbidities, polypharmacy, poor nutrition, and dehydration are particularly significant [9, 10].

Treatment options vary depending on the severity of constipation. Nonetheless, almost half of patients remain dissatisfied with their symptom relief and express concerns about safety, side effects, inconvenience, and taste. [11, 12] Changes in lifestyle behaviors and dietary habits, such as increasing fiber and water intake and exercising, as a first-line treatment, can improve bowel movements. [13, 14] If these measures fail, many individuals turn to osmotic and stimulant laxatives like polyethylene glycol (PEG), lactulose, or other similar osmotic agents. However, these medications are commonly used; the long-term use of them can be associated with adverse complications, such as abdominal pain, bloating, electrolyte imbalance, diarrhea, and drug dependence [1, 15].

Non-pharmacological treatments, also known as complementary and alternative medicine (CAM), encompass a number of approaches for constipation, such as probiotics, acupressure, acupuncture, and abdominal massage, which are widely used for constipation treatment [16, 17, 18]. Herbal medicine, as the most popular modality of CAM, has been considered an effective therapy for constipation [19]. A variety of plants, such as Ficus carica, Malva sylvestris, Foeniculum vulgare Mill., and Cassia fistula L., have been used for the treatment of constipation and have shown positive effects [20, 21, 22, 23].

Traditional Persian Medicine (TPM), a centuries-old form of CAM, recommends various herbal remedies for constipation, whose laxative effects have been confirmed by modern science [24]. Jujube (Ziziphus jujuba L.), also known as Annab in Persian, is a plant that has been widely used for thousands of years in various folk medicines, both as medicine and as food. Its anti-inflammatory, analgesic, antihyperglycemic, antihyperlipidemic, emollient, and laxative properties have been demonstrated in multiple investigations [25, 26]. There are a few clinical trials examining the anti-constipation effects of jujube. Two studies in children found that jujube paste and jujube syrup were as effective as polyethylene glycol in treating FC [27, 28]. Also, a clinical trial on adults showed laxative effects of the liquid extract of jujube [29].

To the authors’ knowledge, no study has evaluated the therapeutic effect of Jujube paste for treating FC in elderly patients. Given the significance of this issue, we conducted this trial to compare the impact of the Jujube oral product with the standard treatment, PEG, for constipation in the elderly, to identify a safe and effective alternative. The primary hypothesis was that jujube paste would improve Rome IV constipation symptoms and stool consistency over 4 weeks.

## Materials and Methods

### Study design

The current study was conducted as a single-blind (data analyst-blinded), randomized, active-controlled, parallel-group clinical trial involving elderly patients diagnosed with FC. Participants and treatment providers could not be blinded because the two interventions differed in appearance, packaging, taste, and administration; therefore, treatment allocation was coded for data analysis. Participants were recruited from the gastroenterology and internal medicine outpatient clinics affiliated with Iran University of Medical Sciences between January 2024 and March 2025. This study was designed as a superiority trial. It was not powered as a non-inferiority or equivalence trial, and no non-inferiority margin was prespecified. This study was carried out following the principles of the Helsinki Declaration and received approval from the Ethics Committee of Mazandaran University of Medical Sciences (Ethics code: IR.MAZUMS.REC.1402.439). It was prospectively registered in the Iranian Registry of Clinical Trials (IRCT ID: IRCT20231022059800N1) on 07/12/2023. All patients gave written informed consent after the purpose of the study was explained.

### Participants

Participants were individuals over 60 years old who had functional constipation based on ROME IV criteria, including the required chronicity of symptoms for at least 3 months with symptom onset at least 6 months before diagnosis, as confirmed by an experienced gastroenterologist. They were mentally alert and able to answer the questionnaire and report their symptoms. Patients with the following criteria were excluded from the study: 1) constipation caused by organic diseases or suspected secondary constipation, including alarm symptoms, suspected colorectal cancer, hypothyroidism, diabetes with suspected autonomic neuropathy, or neurological diseases, 2) involvement in chronic diseases, including cardiovascular, neurological, hematologic, metabolic, severe respiratory, or mental disorders, 3) history of malignant tumors or gastrointestinal conditions like inflammatory bowel disease, 4) kidney or liver failure, 5) history of alcohol use or substance abuse, 6) allergy to jujube or Pidrolax powder, 7) constipation primarily attributed to medications, including opioids, calcium-channel blockers, anticholinergics, iron, calcium supplements, and antidepressants. Patients who could not tolerate the jujube paste or PEG powder, misused them, or were unwilling to continue the intervention were dropped out.

### Sample Size

The sample size was determined using this formula, which is based on the mean and standard deviation of defecation frequency reported in the study by Sepehr et al. [23] With a power (1−β) of 0.80 and a Type I error rate of 0.05, a sample size of 24 per group was initially estimated. The final sample size was increased to 30 per group to account for a potential 20% attrition rate.


n = \frac{\left(z_{1-\alpha/2} + z_{1-\beta}\right)^2 \left(\sigma_1^2 + \sigma_2^2\right)}{\left(\mu_2 - \mu_1\right)^2}

### Randomization and concealment

Eligible patients were randomly assigned to either the Jujube paste or PEG groups using permuted block randomization with a block size of four (ratio 1:1). The random sequence was generated by Random Allocation Software 2.0. The order of the blocks was randomly determined for the Jujube or PEG group, and subjects were assigned based on their admission sequence. A random allocation method was employed, involving sealed, opaque, and sequentially numbered envelopes containing the random sequences.

### Intervention

Participants who met the eligible criteria were allocated to two groups of intervention and control. In the intervention group, patients received jujube paste at the dose of 2 tablespoons (approximately 30 g) of jujube paste, twice daily. On the other hand, patients in the control group were prescribed to dissolve 10-20 grams of PEG powder in warm water or juice and consume daily, preferably 2 hours after breakfast. PEG dosing was flexible within this range and was adjusted according to symptom response and tolerability rather than fixed at the same dose for all participants. Additional laxatives, enemas, probiotics, fiber supplements, or other constipation treatments were not permitted during the intervention.

### Jujube paste and PEG powder preparation and standards

Jujube paste: Dried jujube fruits (Ziziphus jujuba L.) were cut into halves and crushed, then soaked in water for 10 hours. The filtered extract was heated and then filtered. Then, the filtrate was gently concentrated to half its volume to obtain a firm paste. The paste was used as a food-based herbal intervention rather than as a registered medicine. It contained dried jujube fruit and water only; no sugar, sweetener, preservative, coloring, or other additive was added. The prepared paste was dispensed in sealed weekly containers, and participants were instructed to keep it refrigerated throughout the intervention period. Formal stability and microbiological contamination testing were not performed. In the control group, polyethylene glycol powder was used as a laxative. The product, branded Pidrolax, is produced by Sepidaj Pharmaceutical Co., Tehran, Iran, and is available as sachets, each containing 20 grams of PEG 4000.

### Assessment and follow-ups

Initially, participants were asked to complete a general questionnaire covering age, weight, height, gender, marital status, education, medication use, and other medical conditions. After the intervention started, participants were followed for 4 weeks. They were visited weekly to receive enough medication for the next 7 days, and they were also asked to report any side effects and answer any questions they had. Adherence was assessed using self-reported daily recording forms, weekly visits, review of remaining product when available. In addition, patients were called every 3 days to remind them to take their medication and to be addressed for any questions. Participants were asked to maintain their usual diet, fiber intake, fluid intake, and physical activity throughout the study, but these factors were not standardized or measured quantitatively.

### Primary outcomes

The main outcome variables were the severity of constipation and stool consistency. Constipation severity was measured using the Rome IV criteria at the baseline and the end of study. Participants were encouraged to record their data daily on a recording form, either on their own or with help from family members. The criteria on which they made records included the frequency of defecation, straining during bowel movements, the presence of hard or lumpy stool, feelings of anorectal obstruction or blockage, sensations of incomplete evacuation, and the need for defecation maneuvers. Stool consistency was assessed using the Bristol Stool Form Scale (BSFS), a medical tool that classifies stool into seven different types. Type 1 indicates separate hard lumps like nuts, which are difficult to pass and are commonly seen in constipation. Type 2 describes lumpy, sausage-shaped stool, often associated with constipation. Type 3 features a sausage shape with a cracked surface, considered normal. Type 4 is smooth, soft, and sausage- or snake-like, and is also normal. Type 5 consists of soft blobs with clear edges, indicating normal stool. Type 6 is fluffy with ragged edges, composed of a soft mass with an unpleasant odor, usually indicating diarrhea. Type 7 is liquid with no solid material, typically indicating diarrhea [30]. As a primary measure of stool consistency, BSFS was recorded weekly.

### Secondary outcomes

Quality of life was evaluated using the valid and reliable Persian version of the Patient Assessment of Constipation Quality of Life (PAC-QOL) questionnaire, which is recognized as the most specific and valid tool for assessing quality of life in individuals with constipation. It consists of 28 questions about how symptoms impact patients’ daily lives over a two-week period. The questionnaire has four sections: physical discomfort (4 questions), psychosocial discomfort (8 questions), satisfaction with treatment (5 questions), and worries and discomforts (11 questions). All questions are rated on a 5-point Likert scale from 0 to 4. Questions 25-28 are positive statements and are scored inversely. Each participant completed the PAC-QOL both before and after the intervention. Lower scores indicate better quality of life. The patients were also asked about their level of acceptance of the prescribed medication at the end of the study. It was measured on a four-point scale, with 1 indicating “eager consumption,” 2 indicating “consumption,” 3 indicating “reluctant consumption,” and 4 indicating “feeling annoyed.” Moreover, treatment satisfaction among patients in both groups was assessed at the end of the trial using a satisfaction scale, which was a four-point scale with 1 representing “very satisfied,” 2 representing “satisfied,” 3 representing “neutral/not bad,” and 4 representing “not satisfied”.

### Statistical analysis

Data were analyzed using SPSS version 22 software. The Kolmogorov–Smirnov test was employed to assess the normality of the data. Descriptive statistics are reported as mean ± standard deviation (SD) for quantitative variables using an independent t-test, and as frequencies (percentages) for qualitative variables using the Chi-square test. Additionally, an independent t-test was used to compare intergroup changes in constipation severity, quality of life, acceptance, and satisfaction rates. Intra-group changes in constipation severity and quality of life from baseline to the end of the study were analyzed with a paired t-test. Changes in the BSFS score over a 5-point time period were examined using repeated-measures ANOVA. Because baseline education and baseline defecation frequency differed between groups, a post hoc sensitivity analysis for weekly defecation frequency was performed using both change-from-baseline scores and analysis of covariance (ANCOVA). The ANCOVA model included treatment group, baseline defecation frequency, and education category. A p-value below 0.05 was considered statistically significant. The analysis population included participants who completed the 4-week follow-up and was therefore a per-protocol completer analysis. No imputation or intention-to-treat analysis was performed because final outcome data were unavailable for participants who withdrew.

## Results

**Table:** Table1. The baseline data of patients in the two Jujube paste and PEG groups

Baseline data	Jujube paste group(n = 25)	PEG group(n = 26)	P-value
Age	66.32 ± 6.30	65.08 ± 4.74	0.429
BMI	27.99 ± 4.78	27.44 ± 4.42	0.671
Gender			
Male	10 (40.0%)	8 (30.8%)	0.490
Female	15 (60.0%)	18 (69.2%)	
Marital Status			
Single	1 (4.0%)	2 (7.7%)	
0.315			
Married	24 (96.0%)	21 (80.8%)	
Widow/-er	0 (0.0%)	3 (11.5%)	
Education			
Illiterate	1 (4.0%)	4 (15.4%)	
0.004			
Under diploma	6 (24.0%)	15 (57.7%)	
Diploma	9 (36.0%)	6 (23.1%)	
Upper diploma	9 (36.0%)	1 (3.8%)	
Medication intake			
Yes	9 (36.0%)	12 (46.2%)	0.543
No	16 (64.0%)	14 (53.8%)	
Comorbidity			
Yes	11 (44.0%)	12 (46.2%)	0.877
No	14 (56.0%)	14 (53.8%)	

**BMI:** body mass index; **PEG:** polyethylene glycol; **SD:** standard deviation

**Table:** Table2. Comparison of Bristol Stool Form Scale (BSFS) scores between the Jujube paste and PEG groups over time.

Time point	Jujube paste group (n = 25)	PEG group (n = 26)	P value
Baseline (mean ± SD)	1.68 ± 0.90	1.38 ± 0.64	0.185
Day 7 (mean ± SD)	3.88 ± 0.44	4.00 ± 0.40	0.314
Day 14 (mean ± SD)	3.68 ± 0.56	4.38 ± 0.90	0.002
Day 21 (mean ± SD)	3.72 ± 0.89	5.08 ± 1.13	<0.001
Day 28(mean ± SD)	3.88 ± 0.53	4.69 ± 1.01	<0.001

**BSFS:** Bristol Stool Form Scale; **PEG:** polyethylene glycol

**Table:** Table3. Criteria of constipation in two groups of Jujube paste and PEG, in elderly patients with constipation

ROME IV criteria		Jujube paste group n = 25	PEG group n = 26	P-value
Defecation per week (mean ± SD)	Before	3.24 ± 1.80	2.12 ± 1.14	0.012
	After	12.28 ± 2.77	17.58 ± 7.53	0.002
	P-value	<0.001	<0.001	
Straining (%)	Before	91.47 ± 15.67	95.70 ± 10.52	0.265
	After	22.65 ± 17.24	12.96 ± 26.47	0.129
	P-value	<0.001	<0.001	
Lumpy or hard stools (%)	Before	91.56 ± 13.21	95.70 ± 10.52	0.220
	After	16.32 ± 19.90	8.87 ± 26.95	0.268
	P-value	<0.001	<0.001	
Sensation of incomplete evacuation (%)	Before	62.09 ± 38.32	70.77 ± 45.34	0.465
	After	9.17 ± 15.95	5.10 ± 10.80	0.290
	P-value	<0.001	<0.001	
Sensation of anorectal obstruction (%)	Before	26.09 ± 40.99	38.46 ± 49.61	0.338
	After	0.06 ± 2.12	0.05 ± 1.96	0.935
	P-value	0.004	0.0041	
Manual maneuvers (%)	Before	28.76 ± 40.39	46.15 ± 50.84	0.183
	After	4.01 ± 11.64	0.00 ± 0.00	0.098
	P-value	0.002	<0.001	

Note: Values are mean ± SD unless otherwise indicated. Symptom variables represent the percentage of days during the assessment period in which the symptom was present. Defecation frequency is reported as number of bowel movements per week **PEG:** polyethylene glycol; **Rome IV:** Rome IV diagnostic criteria; **SD:** standard deviation.

**Table:** Table4. Quality of life, acceptance, and satisfaction in two groups of Jujube paste and PEG, in elderly patients with constipation.

Jujube paste group n = 25	PEG group n = 26	P-value	
Before (mean ± SD)	44.84 ± 9.07	43.27 ± 11.35	0.589
After (mean ± SD)	15.68 ± 5.80	18.61 ± 3.21	0.032
P -value	<0.001	<0.001	
Satisfaction (mean ± SD)	1.20 ± 0.41	1.58 ± 0.70	0.024
Acceptance (mean ± SD)	1.16 ± 0.37	1.42 ± 0.50	0.039

Note: Lower PAC-QOL scores indicate better quality of life. Satisfaction and acceptance were scored on four-point scales, with lower scores indicating more favorable responses. **PAC-QOL:** Patient Assessment of Constipation Quality of Life; **PEG:** polyethylene glycol; **SD:** standard deviation

**Figure-1:**
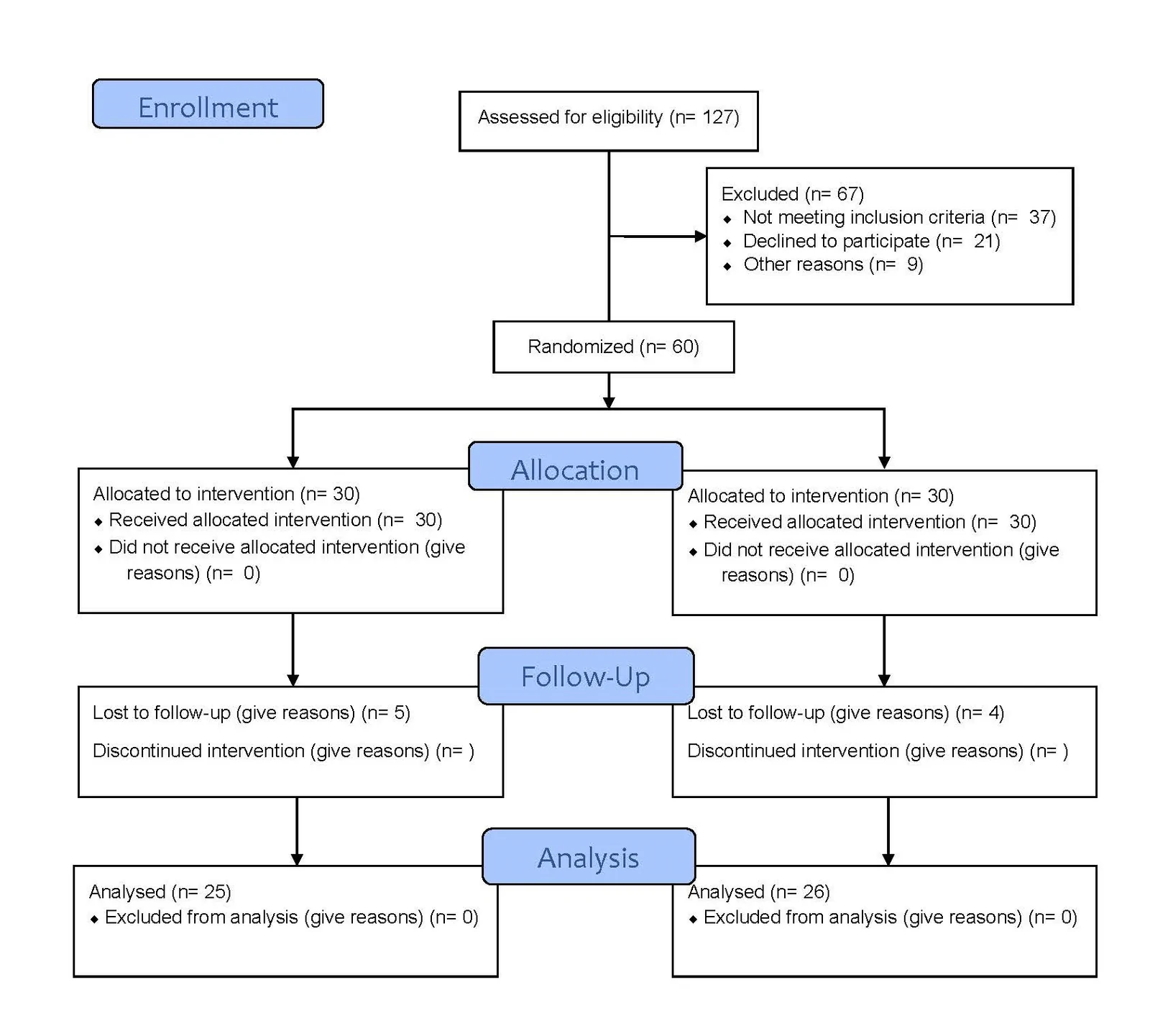
Figure 1. Study Flow Diagram

**Figure-2:**
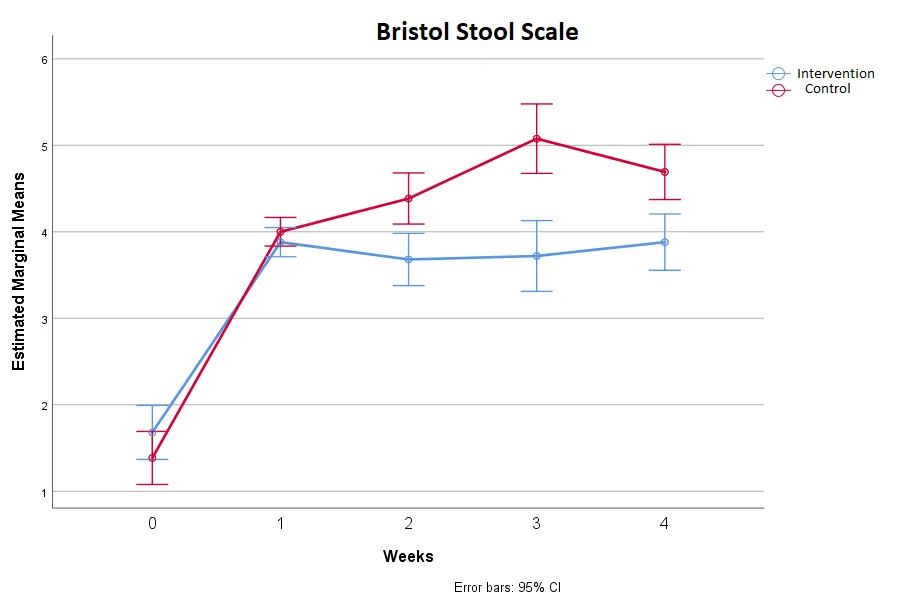
Figure 2. Comparison of Stool Consistency According to the Bristol Stool Form Scale During the Study Period.

A total of 127 elderly patients who visited the gastroenterology and internal medicine clinics with constipation were invited to participate in the study. After assessments, 60 patients who met the eligibility criteria and were willing to participate were recruited for the trial. They were divided into two equal groups: Jujube paste and PEG (30 in each group). Before the final assessment, 5 patients in the Jujube paste group and 4 in the PEG group withdrew due to irregular prescription intake, use of other medications, and experiencing side effects. Consequently, 25 in the Jujube paste group and 26 in the PEG group completed follow-up and were included in the analysis (Figure-1). Demographic characteristics of participants are presented in Table1. The mean age of participants was 66.32 years in the Jujube paste and 65.08 years in the PEG group. About 65% of participants were women. There was no significant difference between groups, except for education (P = 0.004).

### Primary Outcomes

Stool consistency and the severity of constipation, as the primary outcomes of the study, were evaluated using the BSFS and the ROME IV criteria. As presented in Table2, baseline BSFS scores were comparable between the Jujube paste and PEG groups (1.68 ± 0.90 vs. 1.38 ± 0.64, P = 0.185), indicating hard stool consistency consistent with severe constipation in both groups. After 7 days of treatment, BSFS scores increased significantly in both groups, reaching the normal stool consistency range (3.88 ± 0.44 in the Jujube paste group and 4.00 ± 0.40 in the PEG group), with no significant between-group difference (P = 0.314). During the subsequent follow-up, mean BSFS scores were 3.68 ± 0.56, 3.72 ± 0.89, and 3.88 ± 0.53 in the Jujube paste group and 4.38 ± 0.90, 5.08 ± 1.13, and 4.69 ± 1.01 in the PEG group on days 14, 21, and 28, respectively. between-group differences were significant at days 14, 21, and 28 (p-value = 0.002, p-value < 0.001, and p-value < 0.001, respectively), with higher BSFS scores in the PEG group (Figure-2). At baseline, the mean weekly defecation frequency was significantly higher in the Jujube paste group than in the PEG group (3.24 ± 1.80 vs. 2.12 ± 1.14 bowel movements/week, p-value = 0.012). Following the 4-week intervention, defecation frequency increased substantially in both groups, reaching 12.28 ± 2.77 bowel movements/week in the Jujube paste group and 17.58 ± 7.53 bowel movements/week in the PEG group, with a significant between-group difference favoring PEG (p-value = 0.002). Given the baseline imbalance in defecation frequency and education level, treatment effects were further evaluated using both change-from-baseline analysis and ANCOVA. The mean increase in weekly defecation frequency was 9.04 ± 3.51 bowel movements in the Jujube paste group compared with 15.46 ± 7.92 in the PEG group, indicating a significantly greater improvement with PEG (between-group p-value < 0.001). Consistent with these findings, ANCOVA adjusting for baseline defecation frequency and education level demonstrated that the superiority of PEG remained statistically significant at week 4 (adjusted mean difference: 5.20 bowel movements/week; 95% CI, 1.53-8.87; p-value = 0.006). All other constipation criteria, including straining, lumpy stool, sensation of incomplete evacuation, sensation of anorectal obstruction, and manual maneuvers, improved significantly in both groups at the end of the study compared to baseline, while there was no significant difference between the groups (Table3).

### Secondary outcomes

Regarding quality of life, there was no significant difference between the groups at the start of the study (P = 0.589). The mean score changed from 44.84 to 15.68 in the Jujube paste group and from 43.27 to 18.61 in the PEG group, indicating a significant difference between the groups (P = 0.032). Additionally, quality of life improved significantly in both groups after four weeks of treatment (P < 0.001). Finally, satisfaction and acceptance of treatments measured at the end of the study were high in both groups, but significantly greater in the Jujube paste group (p-values = 0.024 for satisfaction and 0.039 for acceptance). (Table4).

## Discussion

In the present study, we compared the effects of Jujube paste and PEG in the treatment of functional constipation in the elderly. The results showed that both treatments significantly improved stool consistency and relieved constipation symptoms up to the normal range. PEG produced a greater increase in weekly defecation frequency, whereas the Jujube paste group showed greater quality-of-life improvement, acceptance, and satisfaction.

Constipation is a common functional gastrointestinal disorder that affects individuals at all ages, especially the elderly [31]. Because of the serious effects of constipation in older populations, finding a safe and effective treatment is essential for them [32]. Laxatives such as lactulose, magnesium hydroxide, lactitol, and PEG are commonly used by the elderly for this condition. PEG is among the most frequently used laxatives to relieve constipation symptoms [31]. The results of the current trial showed that, after a week of PEG consumption, stool consistency, bowel movements, and other Rome IV criteria significantly improved in elderly patients with severe constipation. Consistent with our findings, several trials have demonstrated PEG’s effectiveness in treating constipation [33, 34]. In addition, a long-term trial showed that the effects of PEG are superior to lactulose regarding stool consistency and weekly defecation frequency [35]; however, concerns remain about its adverse side effects and safety. As a result, there is an increasing trend toward using natural therapies, especially herbal remedies [36].

The effectiveness of the herbal formula in treating constipation has been supported by numerous studies. TPM, as a complementary therapy, incorporates several plants with laxative properties, whose effectiveness has been confirmed in randomized clinical trials [24]. For example, the effectiveness of Foeniculum vulgare, Rosa damascena, and Cassia fistula in improving constipation symptoms with high safety has been reported in three clinical trials [22, 23, 37]. Jujube is one of the plants that has been widely used for different conditions, particularly gastrointestinal disorders such as constipation, and is recommended by folk medicines such as TPM and TCM [38, 39].

Based on our data analysis, consuming Jujube paste could significantly improve stool consistency and reduce the severity of constipation. These effects were observed after 1 week of starting the intervention and remained nearly stable through the fourth week. Three clinical trials have previously assessed the impact of Jujube products on constipation; however, none focused on the elderly. In a 12-week randomized, double-blind clinical trial, the effects of Jujube extract were evaluated in adult patients with chronic constipation compared to a placebo [29]. In line with our study, they concluded that Jujube extract considerably decreased the severity of symptoms and transit time [29]. In another study, Keihanian et al. found that Jujube syrup was more effective than PEG in terms of frequency of defecation, while there was no significant difference in terms of fecal consistency [28]. Compared with our study, we observed higher defecation frequency and stool consistency in the PEG group than in the Jujube paste group; however, both were within the normal range. It should be kept in mind that the Keihanian et al. trial was carried out in the pediatric population [28]. Moreover, Yousefi and colleagues conducted a study with a design similar to the present trial, but in children [27]. They compared the efficacy of jujube paste versus PEG for functional constipation and concluded that jujube paste and PEG were similarly effective in improving Rome IV constipation criteria, with jujube paste more effective in reducing stool stiffness [27].

Furthermore, quality of life, acceptance, and satisfaction were also measured as secondary outcomes. Our findings showed that although participants in both groups had a better quality of life when compared with their baseline, it was more pronounced in the Jujube group. In addition, the acceptance and satisfaction rates were considerably higher in the Jujube group in comparison with the PEG group; however, both groups were in a desirable range. In the study by Naftali et al., Jujube extract improved quality of life, whereas no significant change was observed in the placebo group. Additionally, Yousefi and coworkers reported that participants in the Jujube group better accepted intervention than those in the PEG group [27]. Moreover, Keihanian et al. observed higher medication adherence in those who took Jujube syrup compared to the PEG group [28].

Unlike the traditional use of jujube for constipation, its precise mechanism is still not fully understood. Nevertheless, recent research suggests several possible therapeutic benefits. Jujube fruit is high in soluble and insoluble fiber, as well as water-soluble polysaccharides, which become gelatinous in the intestine and help act as a laxative by softening stool [40, 41]. These components also exhibit prebiotic-like activities, which modulate gut microbiota and consequently enhance inflammation and specific metabolic pathways [42, 43]. Intestinal dysbiosis may impair colonic motility, potentially causing constipation [44]. In addition, jujube is rich in flavonoids, saponins, and triterpenes, which have been shown to stimulate intestinal smooth muscle activity and enhance motility [40].

One strength of this study is that it used a natural, food-based intervention to treat constipation in an elderly population that often faces therapeutic restrictions due to their health conditions and polypharmacy intake. However, we acknowledge several limitations: 1) blinding was not possible because the two interventions were markedly different in appearance, packaging, taste, and method of administration; 2) the intervention duration was short, and patients were not followed up after treatment, which limits our understanding of its long-term efficacy and safety; 3) confounding lifestyle factors such as dietary intake and physical activity were not measured, although patients were asked not to change their lifestyle; 4) the small sample size and inclusion of only functionally constipated individuals limit the generalizability of the findings to other populations and conditions; 5) baseline education and defecation frequency differed between groups, which may have influenced symptom reporting, adherence, questionnaire completion, acceptance, and satisfaction; 6) confounding lifestyle factors such as diet, fiber intake, water intake, and physical activity were not measured, although patients were asked not to change their usual lifestyle; 7) medication intake and comorbidity were recorded only as yes/no variables, so the potential effects of polypharmacy, constipating medications, and specific comorbidity categories could not be analyzed; 8) laboratory safety monitoring, including electrolytes, was not performed; 9) formal stability and microbiological testing of the jujube paste were not conducted; 10) the small sample size and recruitment from outpatient clinics affiliated with Iran University of Medical Sciences limit generalizability to institutionalized elderly patients, patients with major comorbidities, and other healthcare settings.

In conclusion, the present study suggests that Jujube, a food-based intervention with high nutritional value, may have beneficial effects on stool consistency, constipation symptoms, and quality of life in elderly individuals with functional constipation. These findings indicate that Jujube could be a promising adjunctive approach for the management of constipation in the short term. However, given the study limitations, including the sample size and study design, larger well-designed randomized controlled trials with longer intervention durations are needed to confirm the efficacy and safety of Jujube and to establish its role in the management of functional constipation.

## Conflict of Interest

The authors declare no conflict of interest.

## AI Disclosure Statement

During the preparation of this manuscript, the authors used ChatGPT, OpenAI company for language editing, grammar improvement, and liboberry.com for reference management. After its use, the authors thoroughly reviewed, verified, and revised all AI-assisted content to ensure accuracy and originality. The authors take full responsibility for the integrity and final content of the published article.
